# Predictors of successful long-term weight loss maintenance: a two-year follow-up

**DOI:** 10.1186/s13030-017-0099-3

**Published:** 2017-06-06

**Authors:** Ryoko Sawamoto, Takehiro Nozaki, Tomoe Nishihara, Tomokazu Furukawa, Tomokazu Hata, Gen Komaki, Nobuyuki Sudo

**Affiliations:** 1 Department of Psychosomatic Medicine, Graduate School of Medical Sciences, Kyushu University, 3-1-1 Maidashi, Higashi-ku, Fukuoka, 812-8582 Japan; 2 School of Health Sciences, Fukuoka, International University of Health and Welfare, Okawa, Japan

**Keywords:** Obesity, Weight maintenance, Cognitive behavioral therapy, Binge eating, Disinhibition, Food addiction

## Abstract

**Background:**

Weight regain is a common problem following weight loss intervention, with most people who seek treatment for obesity able to lose weight, but few able to sustain the changes in behavior required to prevent subsequent weight regain. The identification of factors that predict which patients will successfully maintain weight loss or who are at risk of weight regain after weight loss intervention is necessary to improve the current weight maintenance strategies. The aim of the present study is identify factors associated with successful weight loss maintenance by women with overweight or obesity who completed group cognitive behavioral treatment (CBT) for weight loss.

**Methods:**

Ninety women with overweight or obesity completed a 7-month weight loss intervention. The data of 86 who completed follow-up surveys 12 and 24 months after the end of the treatment was analyzed. Depression, anxiety, binge eating, food addiction, and eating behaviors were assessed before and after the weight loss intervention. Participants who lost at least 10% of their initial weight during the weight loss intervention and had maintained the loss at the month 24 follow-up were defined as successful.

**Results:**

The intervention was successful for 27 participants (31.3%) and unsuccessful for 59 (68.6%). Multiple logistic regression analysis extracted larger weight reduction during the weight loss intervention, a lower disinhibition score, and a low food addiction score at the end of the weight loss intervention as associated with successful weight loss maintenance.

**Conclusion:**

The results suggest that larger weight reduction during the weight loss intervention and lower levels of disinhibition and food addiction at the end of the weight loss intervention predicted successful weight loss maintenance.

**Trial registration:**

Trial registry name: Development and validation of effective treatments of weight loss and weight-loss maintenance using cognitive behavioral therapy for obese patients.

Registration ID: UMIN000006803

Registered 1 January 2012.

URL: https://upload.umin.ac.jp/cgi-open-bin/ctr/ctr_view.cgi?recptno=R000008052

## Background

Weight regain is a common problem following weight loss intervention [1], with most people who seek treatment for obesity able to lose weight, but few able to sustain the changes in behavior required to prevent subsequent weight regain [2]. The identification of factors that predict which patients will successfully maintain weight loss or who are at risk of weight regain after weight loss intervention is necessary to improve the current weight maintenance strategies.

A large number of studies have previously addressed various aspects of this issue. Specifically, binge eating, eating restraint, and disinhibition are among the most studied variables in the context of weight loss and maintenance. However, the results of studies about the association between these variables and long-term weight loss maintenance are inconsistent [3, 4]. This may be partly explained by the fact that most studies only examined factors before the weight loss intervention to identify pre- treatment predictors of long-term weight outcome. Improvement of the eating behaviors that were the targets of these behavioral weight loss interventions might differ because of the weight loss period or weight loss intervention. Therefore, because we felt that eating behavior at the end of our weight loss intervention might be a useful predictor of successful long-term weight maintenance, we investigated both pre- and post-treatment factors.

The concept of food addiction has received much attention in recent years. Previous research has found similarities between addiction to psychoactive substances and excessive food consumption [5]. Gearhardt et al. developed the Yale Food Addiction Scale (YFAS) as an attempt to operationalize the concept of “food addiction” [6]. They assessed YFAS among 81 treatment-seeking, binge eating disorder (BED) patients with obesity and demonstrated that 57% of them had food addiction [7]. Ceccarini et al. also examined 88 patients with obesity, and 34.1% of them were diagnosed after taking YFAS [8] as having food addiction. Although the prevalence of food addiction has been reported to be relatively high in patients with obesity, no studies have assessed the association between food addiction and weight change after a weight loss intervention.

The aim of the present study is to identify factors associated with successful long-term weight loss maintenance after the completion of cognitive behavioral therapy (CBT) for weight loss. To achieve this, we assessed both pre- and post-treatment factors. Specifically, we focused the prognostic value of eating behaviors, including food addiction. We hypothesized that either or both pre- and post-treatment variables of eating behaviors and food addiction might predict successful long-term weight loss maintenance.

## Methods

### Study design

This investigation was a part of a randomized, controlled study that consisted of a two-phase trial that examined two strategies for maintaining weight loss, as previously reported [9, 10]. The weight loss phase provided an intensive program of cognitive behavioral therapy for weight loss that lasted 7 months. Participants who lost at least 5% of their initial body weight during the weight loss phase were eligible for a 3-month program that was provided for the weight maintenance phase. In this phase, the participants were randomized to one of our two weight loss maintenance interventions; CBT including or not including a program to increase adherence to exercise. Follow-ups were done every 6 months for 2 years after the end of the intervention. All of the participants provided informed consent, and the Institutional Review Board of Kyushu University Hospital approved the study protocol.

### Eligibility

All of the participants were women aged 20 to 65 years with a Body Mass Index (BMI) of 25 kg/m^2^ or higher. They were able to understand and complete self-report questionnaires written in Japanese, without assistance, and had no physical impairment that would preclude simple exercise. Those who met the following criteria were excluded from the study: weight loss of more than 5 kg during the previous 6 months, current diagnosis of bulimia nervosa, past history of anorexia nervosa, current pregnancy or breast feeding, planning to become pregnant within the next 24 months, taking any form of medication that would affect body weight, suffering from any health disorder that would affect body weight, receiving nasal continuous positive airway pressure (nCPAP) therapy for obstructive sleep apnea (OSA), currently receiving treatment for a psychiatric disorder, or planning to move within the next 10 months.

### Recruitment and entry

We recruited participants through a local newspaper, the university website, posters in the university hospital and hospitals near the university, and a television program. Those who were interested and potentially eligible were scheduled for an information session to learn more about the study. At the information session, the principal investigator provided the details of the study and answered questions from the participants. Those who met the eligibility criteria were enrolled. After informed consent was obtained, demographic, lifestyle, social status, and health history data were collected.

Binge eating disorder was diagnosed according to the criteria of the fourth edition of the Diagnostic and Statistical Manual of Mental Disorders (DSM-IV) [11].

### Weight loss intervention

Cooper et al. originally developed this CBT program for obesity [12]. We modified it for use in a group therapy program [13] that included the clinical guidelines on obesity of the US National Institutes of Health (NIH) [14], the Action for Health in Diabetes (Look AHEAD) Study [15], and the Diabetes Prevention Program (DPP) [16] as a framework.

Our treatment program was conducted in small groups of approximately 10 people. It consisted of 40 group sessions (30 sessions for the weight loss phase and ten sessions for the weight maintenance phase, each lasting 90 min), and five individual sessions over a 44-week period. The sessions were conducted once a week for the first 34 sessions, with the remaining four held every other week.

The healthcare providers were two physicians who are fully qualified psychotherapists with specialized training in CBT and two certified nutritionists. In the weight loss phase, all participants received a common CBT. They were instructed to keep a daily food diary to track their consumption of all food and drinks and to record their daily number of steps from a pedometer. They were also advised to reduce their dietary intake by 500 cal per day from their caloric intake at the beginning of therapy. The nutritionists checked the nutritional balance of the participants’ diets by examining their food intake diaries and advised the participants about the importance of eating vegetables and reducing the consumption of fatty foods and sweets. The participants were advised to increase their level of physical exercise to a moderate intensity, such as walking 8000–10,000 steps per day by the end of the 7 weeks. Furthermore, a series of lectures on stress management was conducted over eight sessions that included cognitive restructuring, problem solving, and assertion training. During the weight maintenance phase, all participants were instructed to keep their body weight between ± 2 kg of their weight at the end of the weight loss phase. They were randomized to one of two types of CBT in this phase. One was an exercise-with-adherence group and the other was a booster group. Participants who were randomized to the exercise-with-adherence group received CBT that was intended to increase their adherence to exercise, along with nutrition counseling and instruction in stress management. The participants randomized to the booster group received a review and reinforcement of what they had learned in the weight loss phase.

### Anthropometric and body composition measurement

At the entry visit, a fully automatic measuring system (Fancics MX-300) was used to measure height and body weight. Measurement of body weight was done once a week by the patient (on their treatment session day) in the morning in light indoor clothes without shoes. The recorded weight was reported to our staff at each session and follow-up session. BMI was calculated as weight (kg)/height (m^2^)^.^

### Assessment of obesity-related comorbidities

Type 2 diabetes mellitus (T2DM) and Hypertension (HT) were assessed as obesity-related comorbidities. The diagnosis of T2DM was based on the following Japan Diabetes Society criteria: fasting plasma glucose ≥126 mg/dL, symptoms of diabetes plus casual plasma glucose ≥200 mg/dL, or HbA1c ≥6.5% [17]. Patients with a positive history of diabetes and using diabetic medication before entry were also classified as having diabetes despite normal values for fasting plasma glucose or HbA1c while on medication. HT was diagnosed when a participant had been diagnosed with HT before entry and was receiving therapy for HT at entry or when the mean value of two measurements of systolic blood pressure was ≥140 mmHg or diastolic pressure was ≥ 90 mmHg at entry [18].

### Psychological assessment

All participants completed a battery of self-reported psychosocial inventories before and at the end of the weight loss phase.

#### Depression

Depression was evaluated using the Japanese version of the Center for Epidemiologic Studies-Depression Scale (CES-D), for which test-retest reliability and concurrent validity have been thoroughly documented [19]. The CES-D is a 20-item, self-report questionnaire. The scores range from 0 to 60, with a higher score indicating the presence of depressive symptoms [20].

#### Anxiety

Anxiety was evaluated using the Japanese version of the State-Trait Anxiety Inventory (STAI), validated by Nakazato and Nizuguchi [21]. The STAI is a self-report questionnaire consisting of two scales: STAI-1 assesses state-anxiety and STAI-2 assesses trait-anxiety. Each scale consists of 20 items that indicate the presence or absence of anxiety symptoms [22].

#### Binge eating

Binge eating was evaluated using the Binge Eating Scale (BES) [23]. It is a 16-item instrument designed to measure the behavioral and emotional/cognitive symptoms associated with binge eating. We translated the original BES into Japanese and back-translated it. Although it has not yet been standardized, Cronbach’s alpha for the overall scale was 0.867 in the present study, thus we consider it to have good reliability.

#### Eating behavior

Eating behavior was evaluated using the Japanese version of the Three Factors Eating Questionnaire (TFEQ), for which high consistency and construct validity have been confirmed for use with patients who are obese and overweight [24]. The 51-item TFEQ consists of three eating behavior factors; Restraint, Disinhibition, and Hunger [25]. Restraint refers to the tendency of some people to restrict food intake in order to control their body weight. Disinhibition is overconsumption of food in response to a variety of stimuli, such as emotions or alcohol. Hunger refers to food intake in response to feelings and perceptions of hunger.

#### Food addiction

Food addiction was evaluated using the Yale Food Addiction Scale (YFAS). The YFAS is a 25-item tool that assesses addictive-like eating behaviors in the previous 12 months [6]. The YFAS provides two scoring outputs; a total ‘symptom score’ and a ‘diagnosis’ of food addiction. The ‘symptom score’ was used in the present study. We translated the original YFAS into Japanese and back-translated it. Although it has not yet been standardized, Cronbach’s alpha for the overall scale was 0.723 in the present study, thus we consider it to have adequate reliability.

### Follow-up

After the 10-month weight loss and weight maintenance intervention, the participants were instructed to measure and record their body weight every week and to visit the hospital every 6 months for follow-up. In the follow-up sessions, the participants showed the record of their body weight to the investigators. No intervention for weight loss or maintenance was done at the follow-up sessions. Participants who could not attend a follow-up session were instructed to send their body-weight recording sheet to the investigators. For participants who did not attended a follow-up session or send the body weight recording sheet, the investigators phoned or e-mailed to ask their body weight.

### Successful weight loss maintenance

Successful weight maintenance in the present study was defined as having lost more than 10% of the initial weight at the end of the weight loss phase and maintaining at least the 10% loss at the 12- and 24-month follow-ups, following the criteria proposed in the National Weigh Loss Registry study by Wing and Hill [26]. Maintaining a 10% weight loss for it least 1 year is reasonable from a health benefit viewpoint because weight loss of this magnitude can produce substantial improvements in the risk factors for diabetes and heart disease [14]. Furthermore, the findings of the National Weight Control Registry (NWCR) suggested that if individuals can succeed at maintaining their weight loss for 2 years, they can reduce their risk of subsequent regain by nearly 50% [27]. Therefore, in the present study, we followed the participants for 2 years after the intervention and evaluated them as having successful or unsuccessful weight maintenance at both 1 and 2 years after the end of treatment.

### Statistical analysis

The weight loss maintenance pattern was first analyzed by comparing the characteristics of the subjects deemed successful or unsuccessful at the 12-month and 24-month follow-ups. A two-sample *t*-test was used for comparisons between the two groups. The ***χ*** ^**2**^-test or Fisher’s exact test was used to compare nominal data. The variables significant (*P* < 0.05) or near significance (*P* < 0.1) in univariate analysis were then examined as independent variables in direct multiple logistic regression, with successful weight loss maintenance as the dependent variable. The Benjamini-Hochberg procedure was used to control the significance level for increased Type 1 error rate related to multiple significance tests [28]: comparison after multiple logistic regression was done of each individual *p*-value to a corrected *p*-value, (ί/m) *q*, where ί is the rank, m is the total number of tests, and *q* (false discovery rate) is set at 0.1. All statistical analyses were performed with the JMP pro 13.0 software package (SAS Institute Inc., Cary, North Carolina, USA). A *P* value <0.05 was considered significant.

## Results

Of the 119 women with overweight or obesity who participated, 90 completed the 7-month weight loss and weight maintenance intervention. The BW data of one participant who became pregnant, one who withdrew from the study, one who lost contact when studying abroad, and one who received chemotherapy for a malignant disease was lost to follow-up, leaving the data of 86 participants available for analysis. At the 12-month follow-up, 34 subjects (39.5%) were judged as having been successful and 52 (60.5%) as unsuccessful. At the 24-month follow-up, 27 (31.4%) subjects were successful and 59 (68.6%) unsuccessful. The type of weight maintenance intervention did not affect the weight change results at either the 12- or 24-month follow-up (data not shown).

In the analysis of the 12-month follow-up (Table 1), the successful subjects had lost significantly more weight during the weight loss intervention (ΔBW) than had the unsuccessful subjects. The CES-D, BES, disinhibition of TFEQ, and YFAS symptom scores of the successful subjects at the end of weight loss intervention were significantly lower (*P* < 0.05) than those of the unsuccessful subjects. The hunger score of the successful subjects at the end of weight loss intervention was lower than that of the unsuccessful subjects, but it did not achieve significance (P = 0.064). Multiple logistic regression analysis demonstrated that successful weight loss maintenance was associated with larger weight reduction during the weight loss intervention (OR 0.69, 95%CI 0.54–0.83, *P* < 0.001), a lower disinhibition score (OR 0.66, 95%CI 0.46–0.91, P = 0.017), and a lower food addiction score (OR 0.51, 95%CI 0.27–0.89, P = 0.017) at the end of the weight loss intervention. Their significance remained unchanged after the Benjamini-Hochberg procedure (Table 2).

**Table 1:** Univariate analysis of weight loss maintenance at the 12-month follow-up

Variable	Successful	Unsuccessful	*P* value
Number	34	52	
Physical characteristics			
Age (years)	50.6 ± 10.9	46.8 ± 11.7	0.14
Pre BMI (kg/m^2^)	30.7 ± 4.9	31.6 ± 4.7	0.41
Pre BMI ≥ 40 kg/m^2^ (%)	5.9	3.8	0.65
Pre BMI ≥ 35 kg/m^2^ (%)	17.6	17.3	0.97
Pre BW (kg)	75.0 ± 12.1	77.9 ± 12.3	0.30
∆ BW (kg)	−13.3 ± 5.6	−8.4 ± 2.6	<0.0001
Social status (%)			
Level of schooling, >12 years	50.0	46.2	0.73
Employment status, working	58.8	59.6	1.0
Marital status, married	61.8	65.3	0.73
Obesity-related comorbidities (%)			
Type 2 diabetes mellitus	20.6	34.6	0.16
Hypertension	20.6	34.6	0.16
Psychological characteristics (score)			
Pre CES-D	9.7 ± 7.2	12.3 ± 6.5	0.085
Post CES-D	9.7 ± 7.2	15.0 ± 10.1	<0.01
Pre STAI-state	39.9 ± 7.4	41.9 ± 10.6	0.34
Post STAI-state	41.5 ± 10.6	42.5 ± 11.2	0.68
Pre STAI-trait	42.2 ± 10.8	45.2 ± 12.8	0.26
Post STAI-trait	42.2 ± 10.6	42.5 ± 11.2	0.68
Pre BES	12.2 ± 6.9	13.0 ± 6.4	0.56
Post BES	5.6 ± 4.7	8.4 ± 5.8	0.021
Pre dietary restraint (TFEQ)	7.6 ± 3.8	7.6 ± 4.0	0.99
Post dietary restraint (TFEQ)	16.0 ± 3.0	15.0 ± 3.2	0.14
Pre disinhibition (TFEQ)	8.9 ± 3.6	9.8 ± 3.9	0.28
Post disinhibition (TFEQ)	5.7 ± 3.1	7.5 ± 3.6	0.025
Pre hunger (TFEQ)	6.0 ± 2.9	6.4 ± 2.9	0.55
Post hunger (TFEQ)	2.9 ± 2.5	4.1 ± 2.8	0.064
Pre YFAS symptom	1.9 ± 1.5	2.2 ± 1.4	0.27
Post YFAS symptom	1.7 ± 0.97	2.7 ± 1.6	0.001

Quantitative data are shown as mean ± SD. Qualitative data are shown as percentage. Two-sample *t*-test and ***χ*** ^**2**^-test or Fisher’s exact test were used

Intervention was judged successful for subjects who lost at least 10% of their initial weight during the weight loss intervention and had maintained the loss at the12-month follow-up. Intervention was unsuccessful for subjects who did not meet the above criteria at the 12-month follow-up

Pre: data at the beginning of the weight loss intervention

Post: data at the end of the weight loss intervention

∆ BW: Body weight at the end of weight loss intervention minus body weight at the beginning of weight loss intervention

*BMI* Body Mass Index, *BW* Body Weight, *CES-D* Center for Epidemiologic Studies-Depression Scale, *STAI* State-Trait Anxiety Inventory, *BES* Binge Eating Scale, *TFEQ* Three Factor Eating Questionnaire, *YFAS* Yale Food Addiction Scale

**Table 2:** Multiple logistic regression analysis with12-month successful weight loss maintenance as the dependent variable

Independent variable	OR (95% CI)	*P* value	Corrected *P* value
∆ BW	0.69 (0.54−0.83)	<0.001	0.014^*^
Pre CES-D	1.04 (0.93−1.16)	0.52	0.10
Post CES-D	0.93 (0.84−1.00)	0.085	0.057
Post BES	1.16 (0.97−1.41)	0.11	0.071
Post disinhibition (TFEQ)	0.66 (0.46−0.91)	0.017	0.043^*^
Post hunger (TFEQ)	1.24 (0.85−1.84)	0.26	0.086
Post YFAS symptom score	0.51 (0.27−0.89)	0.017	0.029^*^

Pre: data at the beginning of the weight loss phase

Post: data at the end of the weight loss phase

∆ BW: Body weight at the end of weight loss intervention minus body weight at the beginning of weight loss intervention

*OR* Odds Ratio, *CI* Confidence Interval, *BW* Body Weight, *CES-D* Center for Epidemiologic Studies-Depression Scale, *BES* Binge Eating Scale, *TFEQ* Three Factor Eating Questionnaire, *YFAS* Yale Food Addiction Scale

^*^ Significant by corrected *P* value calculated using the Benjamini-Hochberg procedure

In the analysis of the 24-month follow-up (Table 3), the results were similar to those of the 12-month follow-up. The successful subjects lost significantly more weight during the weight loss intervention (ΔBW) than did the unsuccessful subjects. The CES-D, BES, disinhibition, and YFAS symptom scores of the successful subjects at the end of the weight loss intervention were significantly lower than those of the unsuccessful subjects. The hunger score of the successful subjects at the end of the weight loss intervention was lower than that of the unsuccessful subjects, but it did not reach significance (P = 0.095). Multiple logistic regression analysis showed that successful weight loss maintenance was associated with larger weight reduction during the weight loss intervention (OR 0.84, 95%CI 0.72–0.94, *P* < 0.01), a lower disinhibition score (OR 0.68, 95%CI 0.48–0.94, P = 0.028), and a low food addiction score (OR = 0.60, 95%CI 0.34–1.04, P = 0.066) at the end of the weight loss intervention. The former two significance levels remained unchanged after the Benjamini-Hochberg procedure (Table 4).

**Table 3:** Univariate analysis of weight loss maintenance at the 24-month follow-up

Variable	Successful	Unsuccessful	*P* value
Number	27	59	
Physical characteristics			
Age (years)	50.9 ± 10.9	46.6 ± 11.5	0.11
Pre BMI (kg/m^2^)	31.4 ± 4.9	31.1 ± 4.8	0.82
Pre BMI ≥ 40 kg/m^2^ (%)	7.4	3.4	0.59
Pre BMI ≥ 35 kg/m^2^ (%)	14.8	18.6	0.77
Pre BW (kg)	75.7 ± 11.8	77.1 ± 12.4	0.62
∆ BW (kg)	−13.3 ± 5.7	−9.2 ± 3.6	0.0001
Social status (%)			
Level of schooling, >12 years (%)	51.9	45.8	0.60
Employment status, working (%)	44.4	64.4	0.10
Marital status, married (%)	70.4	61.0	0.40
Obesity-related comorbidities (%)			
Type 2 diabetes mellitus	22.2	30.5	0.42
Hypertension	29.6	28.8	0.94
Psychological characteristics (score)			
Pre CES-D	9.7 ± 6.6	12.1 ± 6.8	0.12
Post CES-D	9.6 ± 7.7	14.1 ± 9.7	0.025
Pre STAI-state	39.9 ± 1.8	41.7 ± 1.2	0.43
Post STAI-state	40.7 ± 2.1	42.5 ± 1.4	0.49
Pre STAI-trait	42.1 ± 9.9	45.1 ± 12.8	0.29
Post STAI-trait	42.9 ± 10.8	45.7 ± 11.6	0.29
Pre BES	13.3 ± 7.9	12.4 ± 5.9	0.55
Post BES	5.7 ± 5.2	8.0 ± 5.6	0.076
Pre dietary restraint (TFEQ)	7.6 ± 4.1	7.7 ± 3.8	0.91
Post dietary restraint (TFEQ)	16.0 ± 3.1	15.1 ± 3.2	0.23
Pre disinhibition (TFEQ)	9.6 ± 3.5	9.5 ± 3.9	0.96
Post disinhibition (TFEQ)	5.7 ± 3.2	7.5 ± 3.5	0.024
Pre hunger (TFEQ)	6.4 ± 3.3	6.1 ± 2.8	0.68
Post hunger (TFEQ)	2.9 ± 2.5	3.9 ± 2.8	0.095
Pre YFAS symptom	2.0 ± 1.6	2.1 ± 1.4	0.81
Post YFAS symptom	1.8 ± 1.1	2.5 ± 1.6	0.022

Quantitative data are shown as mean ± SD. Qualitative data are shown as percentage. Two-sample *t*-test and ***χ*** ^**2**^-test or Fisher’s exact test were used

Intervention was judged successful if the subject lost at least 10% of their initial weight during the weight loss intervention and had maintained the loss at 24-month follow-up. Intervention was unsuccessful for subjects who did not meet the above criteria at the end of the 24-months

Pre: data at the beginning of the weight loss intervention

Post: data at the end of the weight loss intervention

∆ BW: Body weight at the end of weight loss intervention minus body weight at the beginning of weight loss intervention

*BMI* Body Mass Index, *BW* Body Weight, *CES-D* Center for Epidemiologic Studies-Depression Scale, *STAI* State-Trait Anxiety Inventory, *BES* Binge Eating Scale, *TFEQ* Three Factor Eating Questionnaire, *YFAS* Yale Food Addiction Scale

**Table 4:** Multiple logistic regression analysis with 24-month successful weight loss maintenance as the dependent variable

Independent variable	OR (95% CI)	*P* value	Corrected *P* value
∆ BW	0.84 (0.72–0.94)	<0.01	0.016^*^
Post CES-D	0.94 (0.87–1.01)	0.11	0.13
Post BES	1.16 (0.97–1.39)	0.10	0.064
Post disinhibition (TFEQ)	0.68 (0.48–0.94)	0.028	0.032^*^
Post hunger (TFEQ)	1.22 (0.84–1.79)	0.30	0.26
Post YFAS symptom score	0.60 (0.34–1.04)	0.066	0.048

Pre: data at the beginning of the weight loss phase

Post: data at the end of the weight loss phase

∆ BW: Body weight at the end of weight loss intervention minus body weight at the beginning of weight loss intervention

*OR* Odds Ratio, *CI* Confidence Interval, *BW* Body Weight, *CES-D* Center for Epidemiologic Studies-Depression Scale, *BES* Binge Eating Scale, *TFEQ* Three Factor Eating Questionnaire, *YFAS* Yale Food Addiction Scale

^*^ Significant by corrected *P* value calculated using the Benjamini-Hochberg procedure

## Discussion

Our multiple logistic regression analysis at both 12 and 24 months after the group CBT weight loss intervention demonstrated that a large amount of weight loss during the weight loss intervention and a low disinhibition score at the end of weight loss intervention was associated with successful weight loss maintenance. A low YFAS symptom score at the end of the weight loss intervention was also associated with successful weight loss maintenance at the 12-month follow-up and weakly associated with successful weight loss maintenance at 24-months.

A meta-analysis showed that persons who lost >20 kg during weight loss intervention maintained significantly more weight loss than those who lost <10 kg [29]. Gow et al. [30] also reported that early (3-month) weight loss was a strong predictor of weight loss at 12-months, which predicted weight loss at a 24-months follow-up of adolescents with obesity. They concluded that this could be because early weight loss identifies people who are more motivated and engaged. Similarly, Vogels et al. [31] reported that % body weight regain after 2 years of a very low-calorie diet (VLDL) was associated with the percent of body fat lost during VLDL. They explained that the result might be because when people lose a large amount of fat, which they might experience through changes in their body shape, they would be more rewarded and willing to keep that lower fat amount. They also explained the result by a fat free mass-sparing effect. Dulloo et al. [32] described this effect, in which the body composition of a given person changes continuously toward a leaner body composition during the course of starvation. Recently, the initial rate of weight loss in the first 2 months predicted weight loss at 4 and 8 years in the Look AHEAD Study, which examined the long-term impact of an intensive lifestyle intervention on cardiovascular morbidity and mortality in overweight or obese persons with type 2 diabetes [33]. Like these, there have been some reports that early weight loss contributes to long-term weight maintenance, whereas some studies demonstrated that more stringent pre-treatment weight expectations were associated with dropout from weight loss intervention or poorer weight outcome [34]. Therefore, it may be useful to do additional intervention for participants who lose a small amount of weight during the weight loss intervention. However, the therapist should not over emphasize at the beginning of the weight loss therapy that participants who lose more weight might be able to successfully maintain their lost weight because it may lead to all-or-none thinking by the participants and may increase the risk of attrition from the therapy. It should always be emphasized that long-term maintenance of the lost weight is much more important for the participants’ health than to quickly lose a large amount of weight.

When patients reduce a high amount of weight, the yoyo-effect or weight cycling often can be problem [35–37]. Weight cycling has been shown to increase the likelihood of future weight gain [35] and has been associated with an increased risk for metabolic syndrome, coronary heart disease, all-cause mortality, and reduced quality of life [36]. In the present study, 10% of the subjects in the unsuccessful group lost more than 10% of their initial body weight during the weight loss phase and had regained weight to more than 97% of their initial body weight after 2 years (data not shown). This pattern of weight change resembles the yoyo-effect or weight cycling (if the pattern is repeated) [35, 37]. Those participants who showed a “yo-yo” pattern of weight change revealed higher scores on the Binge Eating Scale at the end of the weight loss phase than the successful participants who did not (data not shown), consistent with a previous report [38]. Taken together, a large amount of weight loss during weight loss intervention predicts successful long-term weight loss maintenance, however, some participants who were able to lose a large amount of weight but who had a tendency to binge eat at the end of the weight loss intervention might be at risk of weight cycling.

The TFEQ is a widely used measure in obesity research that has often been used to predict weight loss outcome [39, 40]. Specifically, disinhibition has been of particular interest for predicting treatment outcomes. Karlsson et al. [40] found that higher baseline disinhibition predicted weight regain at their 2-year follow-up after a behavioral weight loss treatment. Similarly, Cuntz et al. [41] found that disinhibited eating on completion of an inpatient weight loss treatment program predicted weight regain. In the National Wight Control Registry (NWCR), a registry of successful weight loss maintainers, disinhibition at entry into the NWCR was also shown to predict weight regain 1 year later, and those who regained weight were more likely to report increased disinhibition over the year [42]. However, other studies have failed to find a relation between disinhibition and outcome after weight loss [40]. Niemeier et al. [43] suggested that one reason for the contradictory findings might be the psychometric properties of the disinhibition scale itself. They found that the construct of disinhibition, as measured by the TFEQ, has two factors: internal disinhibition (i.e., eating in response to cognitive and emotional cues) and external disinhibition (i.e., eating in response to environmental cues). Furthermore, they demonstrated in two samples of participants with obesity that lower levels of internal disinhibition were associated with less weight regain after the weight loss intervention, but that external disinhibition was not predictive of weight change. Butryn et al. [44] also reported that participants who experienced the biggest decrease in internal disinhibition during their 3-month meal-replacement-based weight loss program had the most success maintaining their weight loss through the weight maintenance period (from month 4 to 12). The change in external disinhibition was not a significant predictor of weight maintenance. Although we did not evaluate internal or external disinhibition, the disinhibition score at the end of the intervention predicted successful weight loss maintenance, and the scores at the beginning of the intervention did not predict the weight change. In our group CBT, the participants received instruction in cognitive reframing, problem solving techniques, and assertion training and were trained to apply those techniques to their real life [13]. The mean disinhibition scores of our participants improved significantly after the weight loss intervention (*p* < 0.0001, data not shown). If susceptibility to internal disinhibition is causally related to weight regain, long-term obesity treatment outcomes might be improved by spending more intervention time teaching strategies for reducing eating in response to internal cues [42]. In addition, for participants whose disinhibition scores are still high at the end of weight loss intervention, prolonged intervention focused on improving internal disinhibition should be considered.

Food addiction, which was evaluated using the YFAS, has been reported to be associated with depression, negative affect, emotion dysregulation, eating disorder psychopathology, attention-deficit/hyperactivity disorder, and low self-esteem [7, 45]. Few studies have assessed the relation between food addiction and the outcome of weight loss intervention. Lent et al. [46] examined the relation between food addiction assessed by YFAS and the weight and attrition outcomes of adults with overweight and obesity who participated in weight loss intervention. They reported that neither a food addiction diagnosis nor the YFAS symptom score affected weight loss or attrition during their 6-month weight loss treatment, explaining that it was possible that standard weight loss interventions already treat addictive eating behaviors effectively through strategies such as self-monitoring.

Similarly, in the present study, the YFAS symptom score before the weight loss intervention was not associated with weight loss during the intervention or with attrition. In our intervention program, education about the harmful effect of sugar and fatty foods, stimulus regulation, and stress management were included in the weight loss and weight maintenance program, and these could have improved the addictive eating of our participants. Neuroimaging studies, which have recently been used to clarify the relation between neural reactivity and addictive-like behaviors, found that greater reactivity to food cues in the reward- and motivation-associated regions predicted greater future weight gain [47] or a worse outcome in a weight loss program [48]. Although we did not do functional magnetic resonance imaging (fMRI), their results support our findings: participants whose reward systems to foods are highly activated at the end of weight loss intervention are at risk of weight regain afterwards. Taken together, the above indicate that food addiction may exert an impact on weight loss and maintenance in the long-term, although it has less effect on weight loss during the short-term. Thus, it is important to assess food addiction at the end of the weight loss intervention, and additional intervention should be considered for participants who have a high score on the YFAS.

It should be noted that the present study has several limitations. First, our sample size is relatively small. Second, all of the participants were women, and we cannot generalize our results to men with obesity. Third, the Japanese version of the BES and the YFAS that we used are not standardized yet because their validity has not been tested, although they have adequate reliability. Despite these shortcomings, the relatively high follow-up rates and long-term follow-up periods are strengths of our study. They allow us to concretely and accurately show how our participants maintained or regained weight after the intervention. Furthermore, to our knowledge, the present study is the first to evaluate the prognostic value of the YFAS for successful weight loss maintenance after a weight loss intervention. Finally, we evaluated the variables both before and after the weight loss intervention and showed that post-treatment variables are useful for predicting long-term weight outcome. Although none of the pre-treatment factors were predictive of weight change after the weight loss intervention, we gained important hints about the importance of treating the participants for disinhibition and food addiction. In the present study, 31.4% of the subjects were successful at the 24-month follow-up, showing a better outcome than other studies: Cooper et al. reported that 8.2% of their subjects who received 44 weeks of CBT maintained more than 10% weight loss for 2 years after the intervention [2]. In a study by Vogels et al., in which they adopted the same definition of successful weight maintenance as was used in our study, 12.6% of the subjects were successful 2 years after 6 weeks on a very low-calorie diet (VLDL) [29]. However, in the future, we will need to modify and improve our CBT program to focus on factors that were thought to be associated with long-term successful weight loss maintenance in the present study, which will lead to even better results for our patients. . When signs of disinhibition or addiction towards certain types of food (e.g., high fat and sugar) occur during weight loss intervention, consideration should be given to dealing with them early in the program. Also, additional support or alternative options after weight loss intervention could be considered for those persons who are risk of weight gain.

## Conclusion

A larger weight reduction during the weight loss phase and a lower disinhibition score or food addiction at the end of the weight loss phase predicted successful weight loss maintenance. Participants who lose less weight during the weight loss phase and who have a tendency toward disinhibition or food addiction are vulnerable to regaining weight after intervention. Early intervention should be done when these signs are seen, and more intensive or prolonged intervention and frequent follow-up should be considered for such patients after the weight loss phase.

## Abbreviations

BED: Binge eating disorder
CBT: Cognitive behavioral treatment
DES-D: Center for Epidemiologic Studies-Depression Scale
BMI: Body Mass Index
DPP: Diabetes Prevention Program
DSM: Diagnostic and Statistical Manual of Mental Disorders
fMRI: functional magnetic resonance imaging
HEAD: Action for Health in Diabetes
n CPAP: Nasal continuous positive airway pressure
NIH: National Institutes of Health
NWCR: National Wight Control Registry
OSA: Obstructive sleep apnea
STAI: State-Trait Anxiety Inventory
TFEQ: Three Factors Eating Questionnaire
VLDL: Very low-calorie diet
YFAS: Yale Food Addiction Scale
